# Non-Coding RNAs in Stem Cell Regulation and Cardiac Regeneration: Current Problems and Future Perspectives

**DOI:** 10.3390/ijms22179160

**Published:** 2021-08-25

**Authors:** Victor Schweiger, Ena Hasimbegovic, Nina Kastner, Andreas Spannbauer, Denise Traxler, Mariann Gyöngyösi, Julia Mester-Tonczar

**Affiliations:** Department of Internal Medicine II, Division of Cardiology, Medical University of Vienna, 1090 Vienna, Austria; victor.schweiger@student.i-med.ac.at (V.S.); ena.hasimbegovic@meduniwien.ac.at (E.H.); nina.kastner@meduniwien.ac.at (N.K.); andreas.spannbauer@meduniwien.ac.at (A.S.); denise.traxler-weidenauer@meduniwien.ac.at (D.T.); julia.mester-tonczar@meduniwien.ac.at (J.M.-T.)

**Keywords:** circRNA, miRNA, differentiation, stem cells

## Abstract

Although advances in rapid revascularization strategies following acute myocardial infarction (AMI) have led to improved short and long-term outcomes, the associated loss of cardiomyocytes and the subsequent remodeling result in an impaired ventricular function that can lead to heart failure or death. The poor regenerative capacity of the myocardium and the current lack of effective regenerative therapies have driven stem cell research in search of a possible solution. One approach involves the delivery of stem cells to the site of injury in order to stimulate repair response. Although animal studies initially delivered promising results, the application of similar techniques in humans has been hampered by poor target site retention and oncogenic considerations. In response, several alternative strategies, including the use of non-coding RNAs (ncRNAs), have been introduced with the aim of activating and regulating stem cells or inducing stem cell status in resident cells. Circular RNAs (circRNAs) and microRNAs (miRNAs) are ncRNAs with pivotal functions in cell proliferation and differentiation, whose role in stem cell regulation and potential significance for the field of cardiac regeneration is the primary focus of this review. We also address the general advantages of ncRNAs as promising drivers of cardiac regeneration and potent stem cell regulators.

## 1. Introduction

Over the past decade, advances in reperfusion strategies and the ensuing antithrombotic therapies have led to improved outcomes after acute myocardial infarction (AMI). However, despite optimal management, even young individuals are not exempt from the increased morbidity and mortality arising from the resulting myocardial injury [1]. As a consequence of AMI, cardiomyocytes die due to the loss of oxygen and nutrient supply. The loss of functional myocardium results in a reduced contractile function often leading to heart failure (HF) and death [2]. Left ventricular assist devices, total artificial heart systems, and heart transplantation are often the last resort in the treatment of patients with terminal heart failure. However, due to the range of complications associated with these approaches, their technical difficulty, the immense associated costs, the limited availability of donor organs, and the number of specialized centers able to perform these approaches, alternative conservative strategies are badly needed. One approach involves the delivery of stem cells (SCs) to the injury site with the aim of inducing a regenerative response.

SCs are undifferentiated cells that can differentiate into a large majority of different cell types and possess the potential for self-renewal [3]. These distinguishing properties, along with the fact that SCs can be developed and manipulated in culture, have led to great interest in their utilization as therapeutic agents for a variety of different diseases. The basic principle of stem cell therapy is to deliver SCs to the site of injury in order to stimulate a repair response. 

It has been established that the desired effects conveyed by stem cells seem to be carried out either directly, by replacing unhealthy, dysfunctional cells in the damaged tissue, or indirectly. The indirect effects are conveyed through the release of paracrine growth factors, including EGF, VEGF, FGF, SDF-1, TGF-ß, as well as microvesicles containing miRNA, mRNA, ncRNA, and proteins that have the potential to stimulate cardiac regeneration [4,5,6,7]. Several studies demonstrated that coding, as well as noncoding genetic material transferred through microvesicles, is either translated into the corresponding protein or, in the case of noncoding sequences, regulates its respective target in the adjacent cells [8,9,10].

Although it was long believed that the direct mechanism is crucial for inducing the observed benefits, cell retention at the injury site has been proven to be very low [7,11]. Thus the indirect mechanism seems to play a more important role than initially assumed [7,11]. The protective effects induced by the indirect pathways elucidated thus far include the protection of cardiomyocytes from apoptosis and necrosis, the induction of angiogenesis in the infarcted myocardium, the delay of cardiac remodeling, the fusion with resident cardiomyocytes and augmented recruitment of circulating progenitor cells, depending on the applied type of stem cells [12,13,14,15]. 

Besides, even though SCT represents a promising treatment strategy in pre-clinical scenarios, the underlying mechanisms are not fully understood. Clinical trials which applied the principles tested in preclinical studies have only been able to achieve a 5% increase in EF, and the reported results are often unreliable [16]. 

One of the main causes for the mildness of the clinically beneficial effects might be the meager retention at the site of injury and the lack of regenerative potential in the respective tissue [17]. Recent publications have hypothesized that this might be the result of a hostile ischemic and inflammatory environment [18]. Shani et al. reported that the hostile microenvironment of the infarcted myocardium drives MSCs towards a proinflammatory phenotype, leading to a reduced survival rate as well as milder SC paracrine effects [19]. Still, Vagnozzi recently challenged these findings by implying that the inflammatory response after intracoronary SCT may actually be responsible for the beneficial effects [20]. The development of chromosomal abnormalities in the in vitro culture of SCs during processing steps before application, and the differences regarding the proliferative capacity and life span of mesenchymal stem cells between individuals, might also represent limiting factors in clinical studies with SC [21,22,23]. 

In order to improve the disappointing outcomes of stem cell therapy, several mechanisms of modifying the SCs are being tested in preclinical studies. Genetic and non-genetic modifications of SCs like chemical and physical preconditioning or the utilization of biomaterials in order to increase the retention at the injury site might represent mechanisms to further improve the retention, survival, and clinical outcome following SCT. As an example, the therapeutic administration of stem cells with overexpression of SDF-1, IGF, HDF, or MiR-133a after AMI resulted in improvements in cardiac function in preclinical studies on mice [24,25,26,27]. 

However, before stem cell therapy research can move past these limitations, several fundamental aspects need to be elucidated. Particularly, the ideal cell type or a combination of different cells, the optimal dose, delivery, time frame, and frequency of application need to be determined. 

Additionally, individualized SCT approaches are being assessed in order to identify patient’s eligibility for different cell-based regenerative strategies, for instance via the assessment of several biomarkers, in order to improve clinical outcomes [28,29].

## 2. ncRNAs in Cardiac Regeneration

Given the risk factors associated with SCT combined with their poor clinical results, cell-free strategies, such as using conditioned mediums, EVs or small molecules might represent more viable treatment options [30]. These strategies mimic the paracrine effects of SCT and additionally induce the dedifferentiation of resident cardiomyocytes towards proliferating cardiac progenitor cells. Currently, the most promising molecules mimicking the paracrine effect of SCT are ncRNAs.

### 2.1. miRNAs and Their Role in Dedifferentiation and Stem Cell Homeostasis

miRNAs are small non-coding RNA molecules of 20–25 nucleotides. They are post-transcriptional regulatory RNAs that bind to target mRNAs and lead to their transcriptional degradation or suppression. Furthermore, miRNAs play a role in cell differentiation, lineage commitment, proliferation, and apoptosis [27,31].

It may have already been shown, that differentiated as well as proliferating cardiac stem cells (CSCs) highly express various regulatory miRNAs [32]. Several miRNAs might prove to be useful targets for stimulating the regeneration of ischemic myocardium by initiating the dedifferentiation of cardiomyocytes to cardiac progenitor cells or stimulating the activation and proliferation of cardiac progenitor cells. 

Certain miRNAs, such as miR-133, miR-15, and miR-100/99 have been reported to play a major role in controlling the differentiation and expansion of CSCs, as will be elucidated below [32,33,34].

#### 2.1.1. miR-1

The most investigated, cardiomyocyte regulating miRNA is miR-1. The expression of miR-1, with its alleles miR-1-1 and miR-1-2 increases continuously from the early days of embryonic development to adulthood and has been described to enhance both cardiac differentiation as well as exit from the cell cycle, in mammals [35]. miR-1 might convey these effects by reducing the expression of HDAC4, a gene involved in silencing muscle-specific gene expression, or through its direct target Hand2—a transcription factor involved in the development of ventricular cardiomyocytes [36]. The overexpression of either or both of these alleles inhibits cell proliferation in the developing heart of mice and leads to the development of heart failure within embryonic development [36]. miRNA-1-2 deletion in mice either leads to death or thickening of the myocardium as a result of enhanced growth of ventricular cardiomyocytes, probably, depending on the other miR-1 loci [35]. Further, the complete knockdown of miR-1 in mice has been reported to be nonviable, due to severe cardiac dysfunction caused by ventricular septal defects, ventricular dilatation, impaired conduction as well as sarcomere disruption [37]. miR-1 has further been described to worsen oxidative stress and to be overexpressed following AMI in humans, being responsible for a higher amount of arrhythmogenic events in this patient collective [38,39].

#### 2.1.2. miR-15 Family

The miR-15 family includes several miRNAs, such as miR-15a, miR-15b, miR-16-1, miR-16-2, miR-195, and miR-497 and plays a key role in neurodegenerative, cardiovascular, and cancer diseases [40]. Through microarray analyses, Porrello et al. showed that the miR-15 family is highly involved in the postnatal cardiomyocyte mitotic arrest [41]. They also found that the inhibition of the miR-15 family enables cardiomyocyte proliferation beyond the normally observed window of 1–10 days, with an increased number of mitotic cardiomyocytes and the de-repression of checkpoint kinase 1 [41]. Interestingly miR-15 has been shown to be downregulated following AMI in mice [42]. In a murine model of AMI in adult mice, further inhibiting the miR-15 family lead to an alleviated regeneration and enhanced left ventricular function [43]. 

In contrast to the abovementioned targets, which suppress cardiomyocyte regeneration and thus need to be inhibited in order to achieve favorable effects, several miRNAs and miRNA-clusters have been described to directly activate proliferation and increase the regenerative potential in response to cardiac injury. 

#### 2.1.3. miR-133

miR-133a-1 and miR-133a-2 are co-transcribed with miR-1-1/2 and may act as co-players in their function as crucial regulators of skeletal and cardiac muscle development [34,44] miR-133 acts contrary to miR-1 promoting the cardiac progenitor cell state, by repressing SRF [45]. The knockdown of miR-133 leads to increased proliferation of cardiomyocytes in-vitro [46]. While the effects of miR-133 overexpression are miscellaneous, promoting myocardiocyte as well as CPCs survival under oxidative stress by impairing caspase 3 activity and targeting Bim and Bmf, proapoptotic genes, mitigating cardiac fibrosis after myocardial injury in mice, allowing the reprogramming of fibroblasts towards functional cardiomyocytes in-vitro, but decreasing angiogenesis in mice [47,48,49,50]. The overexpression of miR-133 in mesenchymal stem cells (MSCs) has positive effects on their regenerative potential when administered after AMI in mice, with the positive effects most likely driven by miR-133 transfer or an increased release of beneficial paracrine factors [27]. 

#### 2.1.4. miR-99/100

miR-99/100 and Let-7, have been described to be essential in the differentiation of cardiomyocytes [51]. The naturally occurring downregulation of both leads to dedifferentiation of cardiomyocytes and thereby cardiac regeneration, through fntβ and smarca5 in a zebrafish model of infarcted, whereas the administration of both into the infarcted area heavily impairs the given regenerative capability [51]. Moreover, the myocardial overexpression of anti-miR-99/100 and anti-Let-7 in mice after AMI, via lentiviral vectors preserved heart function and reduced myocardial fibrosis [51].

#### 2.1.5. miR-21

miR-21 is a microRNA with distinctive functions in growth, development, proliferation, inflammation, and cardiovascular disease [52].

It has been demonstrated to regulate stem cell migration and proliferation in vitro [53]. In a murine model of AMI, a single injection of miR-21-enriched exosomes induced angiogenesis and preserved the wall thickness and LVF [54]. In a study by Zeng et al.; the intramyocardial administration of stem cells overexpressing miR-21 ameliorated the regenerative response, induced angiogenesis, decreased cardiac fibrosis, and preserved the LVF following cardiac injury induced by anthracyclines [55]. While the role of miR-21 in cardiac fibrosis is still under debate, this study, as well as a study conducted by Olson et al.; could show that the overexpression of miR-21 in cardiomyocytes does not increase cardiac fibrosis following myocardial stress, but likely even decreases the amount of myocardial scarring [54,56]. However, large animal models might further reveal the miR-21′s potential in a translational context. 

#### 2.1.6. miR-218

miR-218 is a microRNA that regulates cardiac stem cell proliferation and differentiation [32]. In a murine model of AMI, the single myocardial administration of miR-218-5p or miR-363-3p enriched exosomes induced angiogenesis decreased cardiac fibrosis and preserved the LVEF [57]. These effects may further be at least partly mediated by the Wnt signaling pathway in the manner of a positive feedback loop [32].

#### 2.1.7. miR-499

miR-499 drives the differentiation of CSCs and thereby diminishes their proliferative potential [31]. It has been shown to target SOX6, a transcription factor that promotes myogenesis [31]. The inhibition of miR-499 and miR-1 with anti-sense RNA molecules disabled the differentiation of CPCs to cardiomyocytes [31]. Furthermore, miR-499 overexpression attenuates hypoxic damage in cardiomyocytes in vitro and apoptosis in vivo and significantly decreases infarct size after AMI in rats [57].

#### 2.1.8. miR-199 and miR-590

miR-199 has also been shown to regulate cardiomyocyte proliferation [58,59]. Sponging miR-199 leads to cardiac hypertrophy in mice [59]. In a large-animal model of pigs, Gabisonia et al. showed that cardiac overexpression of miR-199, via lentiviral vectors, stimulates cardiac regeneration after AMI [58]. However, months of uncontrolled overexpression ultimately led to sudden arrhythmic death in all animals [58]. With the potential of miR-199 overexpression confirmed in large animal models, a controlled expression of miR-199 after AMI may be a promising strategy, provided that better control of miRNA distribution can be achieved by improving miRNA trafficking. However, Lesizza et al. already accomplished that in a rodent model of AMI, but the transition from small animal to large animal models might have difficulties [60].

In a milestone publication by Lesizza et al.; miR-199 and miR-590 were coated by synthetic lipid formulations and injected into infarcted myocardium of mice, which improved the regenerative potential even after a single dose, leading to a preserved LVEF and increase in overall survival of treated mice, compared to the placebo-treated control group [60]. Additionally, all mice survived in the miR-590 injection group [60]. However, because the model of LAD ligation in mice has its difficulties, it is difficult to interpret whether this was due to treatment or milder infarction. However, to mention again, before clinical trials are conducted, large animal models may be inevitable to further clarify the best route, dose, or even sequence of administration.

#### 2.1.9. miR-17-92 Cluster

Chen et al. already demonstrated in 2013, that the miR-17-92 cluster is a crucial regulator of neonatal and adult cardiomyocyte proliferation [61]. Overexpression of miR-17-92 has been shown to induce cardiomyocyte proliferation in vivo and in vitro mediated by the inhibition of Phosphatase and Tensin homolog (PTEN) [61]. In a rodent model of AMI, miR-17-92 overexpression reversed cardiac fibrosis and concomitant cardiac dysfunction following AMI [61].

#### 2.1.10. miR-19

Feng et al. showed in 2019, that the lentiviral overexpression or intramyocardial injection of miR-19a/19b a member of the miR-17-92 cluster decreased myocardial fibrosis, preserved the LVEF, and reduced the mortality after AMI [62]. Importantly, also lipofectamin-mediated systemic miR-19a/19b delivery via injection in the tail vein, improved cardiac function and reduced the infarct size after AMI in mice [62]. These effects might be mediated by targeting PTEN, promoting cardiomyocyte proliferation, and mitigating cardiomyocyte apoptosis following injury [62]. In 2020, Chen et al. showed that the overexpression of miR-19a/19b in MSCs promotes their survival after engraftment in a murine model of AMI [63]. A miR-19a/19b-enriched MSC injection further improved cardiac function after AMI in mice [63]. 

#### 2.1.11. miR-302-367

The miR-302-367 cluster has been shown to be able to maintain progenitor identity [64]. The miR-302 promoter represents a functional target for stem cell factors Sox2, Oct4, and Nanog, and may exert its effects by targeting several downstream targets of the Hippo-pathway [64,65]. In a rodent model, the overexpression of this cluster increased cardiomyocyte proliferation, decreased cardiac fibrosis, and resulted in an improved cardiac function following MI [66]. 

In summary, a handful of miRNAs have been found to regulate the de- or differentiation of cardiomyocytes and thereby affect their proliferative potential. To achieve an adequate proliferation of cardiomyocytes after AMI, at least partial dedifferentiation towards progenitor state might be required, in a way similar to the mechanisms of cardiac regeneration after AMI in zebrafish [67]. Results obtained by Wang et al. speak in favor of this thesis: they show that myocyte proliferation is associated with increased myocyte dedifferentiation in the periinfarct zone of mice, further confirming this thesis in mammals [68].

miRNAs might be potential therapeutic targets for the purpose of boosting the regenerative process of cardiomyocytes after injury (Table 1). However, with the relatively short half-life of miRNAs and a size too large to diffuse across the cell membrane (at about 14 kDa), new ways of trafficking should be explored, however sophisticated lipid packaging might be the key [60]. Moreover, because miRNAs induce various effects and some are even capable of reprogramming cells, it might be inevitable to achieve strict target specificity for the miRNA-containing vesicles.

### 2.2. Linear Long Noncoding RNAs in Stem Cell Regulation and Cardiac Regeneration

Long noncoding RNAs (lncRNAs) are a novel class of regulatory transcripts, larger than 200 nucleotides, with the ability to regulate gene expression at several levels [69]. Currently, up to 548.640 different lncRNAs have been identified [70]. They occur in linear forms or circular forms. However, circular RNAs can also comprise about only 100 nucleotides, making it easier to classify them as their own RNA entity. 

Increasing evidence indicates that linear lncRNAs are differentially expressed across various tissues and act as viable factors in organ development and a wide range of physiological processes, including cell proliferation, apoptosis, cell cycle control, and cell differentiation [70]. Moreover, lncRNAs are emerging as crucial factors in heart development, as well as in several diseases of the heart [71]. In 2014 Matkovich et al. revealed that various lncRNAs show distinctive tissue specificity, and found a number of 321 lncRNAs to be cardiac specific expressed and 52 lncRNAs to be cardiac specific enriched [72]. 

Recently, long non-coding RNAs have been revealed to further act as crucial factors of stem cell regulation and cardiac regeneration [73,74].

#### 2.2.1. Linear Long Noncoding RNAs in Stem Cell Regulation

Studies suggest that several lncRNAs might be involved in the regenerative potential of SCs. However, in most cases, their precise mechanisms of action have not been described yet.

In 2010, Mohamed et al. identified two lncRNAs that are involved in maintaining pluripotency in ESCs, namely AK028326 and AK141205 [75]. Their downregulation decreases the expression levels of several nuclear stem cell factors like Oct4, Sox2, and Nanog [75].

Another lncRNA that regulates stem cell fate is lincRNA-RoR. This lncRNA functions as a ceRNA, sponging miRNAs that target nuclear stem cell factors like Oct4, Sox2, and Nanog [76].

lincRNA-VLDLR is a lncRNA involved in regulating the maintenance of pluripotency [77]. It was identified through its promoter, which is colocalized with stem cell factors Oct4, Sox2, and Nanog [77,78]. However, the exact mechanisms by which lincRNA-VLDLR promotes the maintenance of pluripotency have not been discovered yet.

#### 2.2.2. Cardiac Mesoderm Enhancer-Associated Noncoding RNA (CARMEN)

In 2015, CARMEN was identified as a human super enhancer-associated lncRNA with a distinctive role in regulating the dynamic gene expression during cardiac lineage commitment [79]. Ounzain et al. further revealed that CARMEN is crucial for cardiac specification and differentiation of human cardiac progenitor cells towards cardiomyocytes, as the knockdown of CARMEN in cardiac progenitor cells inhibits heart-specific Fgene expression and the differentiation towards cardiomyocytes [79].

### 2.3. Linear Long Noncoding RNAs in Small Animal Models

#### 2.3.1. ECRAR

ECRAR is a linear lncRNA that is abundantly expressed in the developing heart [80]. Its overexpression promotes rat cardiomyocyte proliferation in vitro as well as in vivo [80]. Interestingly, the overexpression of ECRAR also decreases the secretion of hypertrophy-related markers by cardiomyocytes [80]. In a rodent model of AMI, the overexpression of ECRAR via an adenoviral vector ameliorated the regenerative response, inducing angiogenesis and preserving the LVEF [80]. While in contrast, the knockdown of ECRAR impaired cardiac regeneration after AMI [80]. These effects might be mediated by ERK1/2, as ECRAR triggers the translocation of ERK1/2s into the nucleus [80].

#### 2.3.2. CAREL

CAREL is a linear lncRNA that has been found to be highly expressed in neonatal mouse hearts, particularly from the 7th day on, when the proliferative capacity of cardiomyocytes gets naturally compromised [81]. The constitutive overexpression of CAREL mitigates the proliferative potential of mural neonatal cardiomyocytes and thereby significantly increases their size [81]. The knockdown of CAREL, via the administration of adenoviral short hairpin RNAs (ad-shCAREL) into the site of injury after AMI, promoted the proliferation of cardiomyocytes and thereby significantly reduced scar size, leading to a preserved LVF [81]. This might be achieved by acting as an ceRNA, sponging miR-296 and thereby inducing the expression of Trp53inp1 and Itm2a [81].

#### 2.3.3. Silent Information Regulator Factor 2 Related Enzyme 1 (Sirt1) Antisense

In 2018 Li et al. identified silent information regulator factor 2 related enzyme 1 (Sirt1) antisense as a novel regulator of cardiomyocyte proliferation and cardiac regeneration [82]. The overexpression of Sirt1 antisense in mural neonatal cardiomyocytes promotes their proliferative potential in vitro and in vivo [82]. In a mural model of AMI, the AAV-9 mediated overexpression of Sirt1 preserved cardiac function and further resulted in an increased survival rate after 4 weeks [82]. These effects may be mediated by increasing Sirt 1 mRNA expression and stability [82].

#### 2.3.4. NR_045363

The cardiomyocyte-specific lncRNA NR_045363 is a linear lncRNA dynamically expressed during cardiac development, with distinct regenerative properties [83]. The knockdown of NR_045363 in embryonal cardiomyocytes diminished their proliferative potential [83]. The intramyocardial administration of an AAV9 containing NR-045363, subsequently to AMI in neonatal mice preserved the LVF and decreased cardiac fibrosis compared to the control-injected group [83]. The proliferative effects may be exerted via the miR-216a/JAK2-STAT3 pathway, sponging miR-216a [83]. Furthermore, NR_045363 might also regulate cardiomyocyte apoptosis, via the activation of the p53 signaling pathway [84]. 

#### 2.3.5. Cardiomyocyte Proliferation Regulator (CPR)

CPR is a lncRNA that is highly expressed in mural myocardium as well as other organ-related tissues and has been demonstrated to downregulate cardiomyocyte proliferation in vitro [85]. In CPR knockout mice, the proliferative capacity of cardiomyocytes was sustained throughout the observational timeframe and no decrease in cardiac function could be detected [85]. The constitutive overexpression of CPR in mice didn’t trigger any morphological nor functional alterations, compared to WT mice [85]. However, cardiac hypertrophy markers were significantly increased [85]. The AAV9 mediated overexpression of CPR after AMI preserved the LVEF and decreased the amount of myocardial fibrosis in neonatal mice [85]. Moreover, in adult CPR knockout mice the regenerative response after AMI was significantly improved [85]. These effects might be exerted by the inhibition of CPR-initiated repression of MCM3 [85]. 

#### 2.3.6. Wisp2 Super-Enhancer-Associated RNA (Wisper)

The lncRNA Wisp2 super-enhancer–associated RNA (Wisper) is a lncRNA conserved in humans, that correlates with collagen content and cardiac fibrosis [86]. It has been described to regulate the actions of cardiac fibroblasts in vitro and in vivo [86]. It is transcribed from a cardiac-specific super-enhancer region and was demonstrated to be highly upregulated in the infarct border zone after MI in mice [86]. Wisper regulates proliferation, migration, and apoptosis via the expression of pro-fibrotic factors, specifically in cardiac fibroblasts [86]. In a murine model, the GapmeR-mediated knockdown of Wisper subsequently to AMI decreased the infarct size, perturbated cardiac fibrosis, and preserved cardiac structure and function [86]. Wisper directly interacts with TIA1-related protein (TIAR) to regulate the alternate splicing, and thereby the expression of lysyl hydroxylase 2 (LH2), which is associated with tissue fibrosis and viable for collagen crosslinking [86,87]. 

In conclusion, studies suggest that several lncRNAs are involved in regulating cardiac regeneration and stem cell regulation (Table 2). However, with a recent study suggesting that only 1% of lncRNAs have been identified yet, their potential therapeutic role in the field of cardiac regeneration might expand in the upcoming years. Especially, as their linear form, and thereby easier production and trafficking, compared to circRNAs might be an advantage at the current state of knowledge, regarding RNA-based therapeutic strategies.

### 2.4. CircRNAs Regulate SC Fate

CircRNAs are a class of ncRNAs that have recently reentered the scientific spotlight. They are involved in thousands of physiological and pathophysiological regulatory networks and are processed by a wide array of different cell types through a process called backsplicing. The large diversity and relative abundance are illustrated by the fact that approximately 9% of all genes in the human and mouse heart have been observed to produce circRNAs [88]. Furthermore, some circRNAs have a higher transcript abundance compared to their linear counterparts. CircRNAs have longer half-lives compared to other RNA molecules because of their resistance to exonucleases [89,90]. CircRNA lengths span between less than 100 base pairs and several kilobases [91]. Their mechanisms of action mainly involve acting as vehicles for interactions between proteins or as miRNA sponges. 

Ruan et al. found a total of 226 circRNAs that were either upregulated or downregulated during the differentiation of human umbilical cord-derived MSCs (huMSCs) [92]. Their research also underlined the involvement of some of these circRNAs in organogenesis and regeneration, as some of the most highly regulated circRNAs appeared to be related to cell proliferation pathways, for example, the Wnt or Hedgehog signaling pathways, as a result of a gene ontology analysis [92].

#### 2.4.1. CircFoxP1

CircRNAs are also involved in stem cell homeostasis. CircFoxP1, the circular transcript of the FOXP1 gene, has been found to be one of the most highly upregulated circRNAs in MSCs, however, the upregulation markedly decreases following the differentiation of those cells [93]. Moreover, silencing circFOXP1 through siRNA markedly reduces MSC proliferation and downregulates the expression of MSC surface markers [93]. One of the underlying mechanisms of action represents its role as a competing endogenous RNA (ceRNA), interacting with miRNAs such as miR-17-3p and miR-127-5p, particularly by sponging them. The circFOXP1-miR-17-3-p/miR-127-5p axis has been shown to be involved in EGFR- and non-canonical Wnt signaling pathways [93]. Non-canonical Wnt signaling is a downstream pathway of Myc and participates in both MSC proliferation and self-renewal [94].

#### 2.4.2. CDR1as

Another circRNA that regulates MSC homeostasis, in particular, is CDR1as. A study by Yang et al. revealed that CDR1as is able to maintain pluripotency in MSCs [95]. The knockdown of CDR1as decreased the proliferation and induced apoptosis of MSCs [95]. The authors postulated that these effects may be triggered by a decreased expression of PCNA, Bcl-2, as well as an increased expression of caspase 3 and 9 [95]. The CDR1as knockdown also leads to a reduced expression of Oct4, Sox2, and Nanog, factors well known for their indispensable role in determining the stem cell fate [96,97,98]. A study in pigs discovered a protective effect of CDR1as with regards to cardiac fibrosis, which it achieves by acting as a ceRNA, interacting with miR-7 [99]. Particularly, Mester-Tonczar et al. demonstrated, that elevated tissue levels of CDR1as in the infarcted area of pig myocardium following AMI correlated significantly with LV and right ventricular EF, LV stroke volume, and negatively with infarct size [99]. However, an animal model of overexpressing CDR1as after AMI in the injured area is still lacking so far.

#### 2.4.3. CircBIRC6

CircBIRC6 and circCORO1C play crucial roles in hESC pluripotency [100]. The inhibition of either of these circRNAs leads to a decreased expression of the Yamanaka factors NANOG, OCT4, KLF4, and MYC in SCs [100]. The effects of circBIRC6 seem to be conveyed through the interaction with miR-34a and miR-145, whereby circBIRC6 sponges the two miRNAs and thereby suppresses their potential to induce differentiation of ESCs [100]. The underlying pathway of circCORO1C, on the other hand, has not been elaborated yet [100]. Furthermore, miR-34 inhibition enhances human cardiac progenitor cell proliferation in-vitro, but in order to replicate the therapeutic effects in humans, further evaluations in in-vivo models of myocardial infarction would be required [101].

### 2.5. CircRNAs in Small Animal Models of Myocardial Regeneration

#### 2.5.1. CircHipK3

CircHipk3 is a circRNA that Yang et al. demonstrated to be highly expressed in MSCs [95]. While its exact role in SC regulation has not been described yet, Si et al. found that circHipk3 is highly expressed in the neonatal hearts of mice and seems to be highly conserved between species [102]. CircHipk3 is regulated by Gata4, a marker known to be involved in cardiomyocyte proliferation, and its effects are thought to be executed by increasing the stability of Notch1, an important regulator of cardiomyocyte proliferation [102]. The overexpression of circHipk3 in the infarcted myocardium of mice led to the proliferation of cardiomyocytes, preserves heart function, and decreases myocardial fibrosis, compared to the control group [102]. In addition, the effects were mediated by sponging miR-133, and thereby induction of angiogenesis via the proliferation of endothelial cells [102]. In conclusion, CircHipk3 is involved in a range of beneficial mechanisms that contribute to the regeneration of the ischemic myocardium, which makes it a promising therapeutic target.

#### 2.5.2. CircCDYL

CircCDYL is a circRNA, that plays a role in the regulation of cardiomyocyte proliferation [103]. The overexpression of circCDYL via adenoviral vectors in a rodent model of AMI led to an increased LVEF compared to the control group overexpressing si-circCDYL via an adenoviral vector [103]. These effects may, at least partly be exerted by acting as a ceRNA, sponging miR-4793-5p, that is involved in the regulation of APP [103].

#### 2.5.3. CircNfix

While the overexpression of the abovementioned circRNAs improves the physiological regenerative effects, circNfix appears to have predominantly negative effects. CircNfix is supposed to have a two-pronged mechanism of action—by stimulating the Ybx1 ubiquitination on the one hand and acting as a sponge for miR-214 on the other [104]. CircNfix overexpression decreased cardiomyocyte proliferation, while its knockdown increased proliferation and angiogenesis and further deteriorated the ubiquitin-mediated degradation of cardiomyocytes after AMI in mice [104]. 

#### 2.5.4. Circ0060745

Circ0060745 is a circular RNA that is overexpressed in murine myocardium, particularly cardiac fibroblasts following AMI [105]. In a rodent model of AMI, the knockdown of circ0060745 via lentiviral vectors inhibited cardiomyocyte apoptosis, decreased myocardial fibrosis, and preserved the LVEF [105]. In vitro experiments further revealed that circ0060745 knockdown suppresses the activation of NF-kB, decreases the expression levels of Interleukin-6 (IL-6), IL-12, IL-1ß, and Tumor necrosis factor α (TNF-α) under hypoxic stress in mural cardiac fibroblasts [105].

#### 2.5.5. CircNCX1

A study published in 2018 elucidated the proapoptotic role of CircNCX1 on cardiomyocytes following hypoxic injury [106]. In a model of murine ischemia-reperfusion injury, the knockdown of circNCX1 by shRNAs preserved the LVEF and decreased myocardial fibrosis by acting as a miR-133 sponge, a microRNA that has already been demonstrated to enhance the survival of cardiomyocytes and cardiac progenitor cells after AMI and to promote cardiac progenitor cell state [50,106].

#### 2.5.6. CircFNdc3b

The overexpression of CircFNdc3b via lentiviral vectors leads to improved cardiac regeneration, preserving the LVEF and amount of cardiac fibrosis after AMI by inhibiting cardiomyocyte apoptosis and inducing angiogenesis in mice [107]. These beneficial effects may be mediated, at least in part, by the FUS-VEGF axis [107]. 

#### 2.5.7. Circ0001273

In 2020, Li et al. found that circ0001273 is abundantly expressed in MSCs and transported in MSC-derived exosomes, respectively to its expression levels in the MSCs [108]. In an AMI model in rats, injecting MSC-derived exosomes containing si-circRNA001273 into the infarcted area underperformed the regenerative effects induced by injecting exosomes derived from unmodified MSC [108]. Further, the beneficial effects of this circRNA have been shown to be mediated by promoting cardiomyocyte cell survival after AMI [108].

In conclusion, several circRNAs with roles in stem cell regulation have been discovered, whose effects in this regard seem to be mainly conveyed by acting as ceRNAs, inhibiting miRNAs, interacting with proteins, and increasing the expression of crucial nuclear stem cell factors. The effects of circRNAs thereby exceed an isolated increase in the proliferation of cardiomyocytes, inducing angiogenesis and promoting cardiomyocyte survival (Table 3). Therefore, circRNAs may represent promising therapeutic targets. 

## 3. Current Difficulties regarding ncRNAs-Based Therapies

Whereas miRNA-based approaches have already been implemented into the clinical setting, the clinical use of circRNA-based therapeutic approaches remains challenging [109].

Although a knockdown of circRNAs via short hairpin RNA molecules seems feasible as demonstrated in small animal models, inducing overexpression of the respective circRNAs could prove to be challenging due to several issues complicating the task [105,106].

The synthetic mass production of circRNAs might prove difficult, as strand-displacing RT enzymes can falsely create long cDNAs [109,110]. These consist of concatemers, which are toxic on the one hand, and can also function as substrates for linear splicing, making their in vivo effects difficult to predict [109,110]. In 2018, Jost et al. designed and produced a circRNA molecule, that can act as a sponge for miR-122 in vitro and might represent an effective therapeutic for HCV-mediated liver injury in vivo [111]. In 2020, Breuer and Rossbach published a protocol for the design, production, and purification of synthetic circular RNAs [112]. Both protocols include a step for the purification of linear transcripts. However, validating the level of purification effectiveness will be crucial in ensuring that no unpredicted adverse effects occur in patients. 

The stability of circRNAs is higher compared to linear RNA species, which might affect their bio-distributional properties and thereby affect their therapeutic ability. CircRNAs are large and insufficiently hydrophobic for passive diffusion, and thus require specific ways of administration or overexpression [109,113]. Sophisticated ways of RNA trafficking might be needed in order to reach an adequate distribution and to rule out side-effects due to the cumulation of the agent in other tissues. Since circRNAs have only recently come back into the scientific spotlight, the best route for their delivery has not yet been identified. However, as Holdt et al. have already elaborated, developing strategies for their specific distribution might directly benefit from the existing experience with RNA trafficking via lipid carriers or functionalized nanoparticles [109].

Another strategy for achieving sufficient distribution of circRNAs in the targeted tissue is promoting their endogenous expression via adenoviral vectors. However, while adenoviral delivery of genetic material seems to be very promising, there are still issues surrounding their use in humans. These issues concern their contamination with helper viruses, previous immunization against adenoviruses, as well as tissue damage and inflammation [114]. However, solutions for these issues are currently targeted and are elaborated in this cited review by Lee et al. [114]. 

## 4. Conclusions

This review outlines the current advances in research on, advantages and obstacles of the use of ncRNAs and SC regulation. Here we discussed the role of ncRNAs in SC regulation and cardiac regeneration. Although the underlying mechanisms of the effects of most of these molecules have not yet been completely elucidated, they represent promising therapeutic targets with beneficial effects comprising reverse cardiac remodeling, inducing angiogenesis, the promotion of cardiomyocyte survival, and the expansion of cardiac progenitor cells. Although certain mechanisms of RNA trafficking seem very promising, like the single intramyocardial injection of distinct RNA species or the lipofectamine-mediated delivery of RNA species via intravenous administration, in case of distinct RNA specificity to a certain tissue, large animal models may be required before their translation into humans. 

## Figures and Tables

**Table 1:** Summary of miRNAs considered to be involved in stem cell regulation and cardiac regeneration.

miRNA	Function	Potential Targets	Animal Models	Mechanism	In Vivo Effects
miR-1	Differentiation into Cardiomyocytes and exit of the cell cycle	HDAC4, Hand2	Mice	Overexpression	Worsens oxidative stress in the context of AMI and favors arrhythmogenic events
miR-499	Differentiation of cardiomyocytes	sox6	Mice	Local administration of agomiR-499	Decreases fibrosis and attenuates apoptosis of cardiomyocytes after AMI
miR-99/100	Differentiation of cardiomyocytes	fntß, smarca5	Zebrafish, mice	Underexpression	Decreases fibrosis, preserved LVEF after AMI
miR-15 Family	Cell cycle arrest of cardiomyocytes, promoting cardiac progenitor survival	Checkpoint kinase 1	Mice	Underexpression	Decreases infarct size and cardiac remodeling and increases LVEF after AMI
miR-133a-1/2	Differentiation of cardiomyocytes, promoting cardiac progenitor survival, reprogramming towards cardiomyocytes	RhoA, MAPK, TGFß/Smad, Pi3K/Akt, Bim, Bmf, etc.	Zebrafish, mice	Administration of MSC overexpressing miR-133	Decreases fibrosis, attenuates apoptosis, enhances survival of progenitor cells after AMI
miR-199	Cardiomyocyte proliferation, important role for the Excitation generation and conductive system of the heart	cd151, mTOR, and approximately 641 other genes	Mice, pig	Overexpression or local administration	Preserves LVEF after, increases survival of pigs after AMI
miR-17-92 cluster	Cardiomyocyte proliferation	PTEN	Mice	Overexpression	Decreases fibrosis, preserves LVEF after AMI
miR-21	Stem cell proliferation and migration		Mice	Overexpression or local administration	Induces angiogenesis, decreases fibrosis, and preserves LVEF after anthrazycline induced cardiac injury
miR-218	Cardiac stem cell proliferation	Wnt	Mice	Local administration	Induces angiogenesis, decreases fibrosis, and preserves LVEF
miR-302-367 cluster	Maintaining progenitor identity	sox2, oct4, nanog Hippo-pathway	Mice	Overexpression	Decreases fibrosis, preserves LVEF after AMI
miR-590	Currently unknown	Approximately 641 other genes	Pig	Local administration	Preserves LVEF and increases survival of pigs after AMI

LVEF: Left ventricular ejection fraction, AMI: acute myocardial infarction.

**Table 2:** Summary of linear lncRNAs considered to be involved in stem cell regulation and cardiac regeneration.

lncRNA	Function	Potential Targets	Animal Models	Mechanism	In Vivo Effects
AK028326, AK141205, lincRNA-RoR, lincRNA-VLDLR	Maintaining pluripotency of SC	Nuclear stem cell factors			
CARMEN	Differentiation to cardiomyocytes	several			
ECRAR	Proliferation of cardiomyocytes	ERK1/2	Rats	Overexpression	Decreases fibrosis, preserves LVEF, and induces Angiogenesis after AMI
CAREL	Proliferation of cardiomyocytes	Sponging miR-296—inducing Trp53inp1 and Itm2a	Mice	Knockdown	Decreases fibrosis, preserves LVEF after AMI
Sirt1 antisense	Proliferation of cardiomyocytes	Sirt1 expression and stability	Mice	Overexpression	preserves LVEF, decreases mortality after AMI
NR_045363	Proliferation of cardiomyocytes, promoting cardiomyocyte survival	Sponging miR-216a, p53	Mice	Overexpression	Decreases fibrosis and preserves LVEF after AMI
CPR	Proliferation of cardiomyocytes	MCM3	Mice	Overexpression	Decreases fibrosis, preserves LVEF after AMI
Wisper	Regulates cardiac Fibroblast actions	Interacts with TIAR, increases LH2	Mice	Overexpression	Decreases fibrosis, preserves LVEF after AMI

LVEF: Left ventricular ejection fraction, AMI: acute myocardial infarction.

**Table 3:** Summary of circRNAs considered to be involved in stem cell regulation and cardiac regeneration.

circRNA	Function	Potential Targets	Animal Models	Mechanism	In Vivo Effects
CircFoxP1	MSC proliferation	sponging miR-17-3p and miR-127-5p, EGFR and non-canonical Wnt signaling	Human		Not elaborated yet
CDR1as	Maintaining pluripotency of MSC	oct4, sox2, nanog, PCNA, Bcl-2, caspase 3, caspase 9	pig		Not elaborated yet
circBIRC6	Maintaining pluripotency of ESC	Sponging miR-34a, miR-145, oct4, klf4, myc, nanog			Not elaborated yet
circCORO1C	Maintaining pluripotency of ESC, proliferation of progenitor cells	oct4, klf4, myc, nanog	Human		Not elaborated yet
circHipk1	Cardiomyocyte proliferation, endothelial proliferation	Sponging miR-133, Notch1	Mice	overexpression	Decreases fibrosis, preserves LVEF after AMI
CircNfix	Regulates cardiomyocyte proliferation, increasing angiogenesis	Sponging miR-214, Ybx1	Mice	overexpression	Decreases fibrosis, deteriorates degradation of cardiomyocytes after AMI
CircCDYL	Cardiomyocyte proliferation	Sponging miR-4793-5p	Mice	overexpression	Preserves LVEF after AMI
CircFNdc3b	Promoting cardiomyocyte survival, increasing angiogenesis	FUS-VEGF	Mice	overexpression	Decreases fibrosis, preserves LVEF after AMI
Circ0001273	Inhibits cardiomyocyte apoptosis	Not elaborated yet	Rats	injecting exosomes containing circ-0001273	Preserves LVEF after AMI
Circ0060745	Promotes cardiomyocyte apoptosis	NFkB, IL-6, IL-12, IL-1ß, TNF- α	Mice	knockdown	Induces angiogenesis, decreases fibrosis, and preserves the LVEF after AMI
CircNCX1	Promotes cardiomyocyte apoptosis	Sponging miR-133	Mice	knockdown	Decreases fibrosis and preserves LVEF after ischemia/reperfusion injury

LVEF: Left ventricular ejection fraction, AMI: acute myocardial infarction.
